# Genome Sequence of a Promising Hydrogen-Producing Facultative Anaerobic Bacterium, *Brevundimonas naejangsanensis* Strain B1

**DOI:** 10.1128/genomeA.00542-14

**Published:** 2014-06-05

**Authors:** Haijia Su, Ting Zhang, Meidan Bao, Yixin Jiang, Yu Wang, Tianwei Tan

**Affiliations:** Beijing Key Laboratory of Bioprocess, Beijing University of Chemical Technology, Beijing, China

## Abstract

*Brevundimonas naejangsanensis* strain B1 is a newly isolated, facultative anaerobic bacterium capable of producing hydrogen with high efficiency. Here, we present a 2.94-Mb assembly of the genome sequence of strain B1, which may provide further insights into the molecular mechanism of hydrogen production from bioresource.

## GENOME ANNOUNCEMENT

Aimed at addressing the world’s energy and global warming crises, extensive efforts have been devoted to the production of clean and renewable energy resources (1). Hydrogen (H_2_) is regarded as a promising energy resource because it has a specific enthalpy content that is more than twice that of methane and three times that of coal, and water is the sole product when H_2_ is combusted to release energy (2). Biological H_2_ production by microorganisms from biomass has become increasingly attractive, as it is renewable and often relatively cheap to maintain (3). A large number of microorganisms are capable of producing H_2_, whereas strict anaerobic conditions are usually required for high-yield H_2_ production (4–6). Some facultative anaerobes can produce H_2_ in quantities comparable to those produced by strict anaerobes. Moreover, their ability to survive in the presence of O_2_ offers an additional advantage during the initial stage of anaerobic H_2_ production (4).

*Brevundimonas naejangsanensis* strain B1 is a facultative anaerobic, acidogenic H_2_ producer newly isolated from the activated sludge of an anaerobic digestion reactor. Strain B1 is able to ferment sugars into hydrogen with high efficiency (7, 8). When *B. naejangsanensis* strain B1 was co-cultured with another starch-utilizing H_2_ producer, *Bacillus cereus* strain A1, a higher H_2_ production efficiency and a shift in end products from ethanol and propionic acid to acetic acid and butyric acid were obtained (7, 8). However, the mechanisms need to be further characterized. Genome sequencing of *B. naejangsanensis* strain B1 will be of great help in this regard.

Here, we present the ﬁrst draft genome sequence of *B. naejangsanensis* strain B1, obtained by using the Illumina HiSeq 2000 system, which was performed by the Chinese National Human Genome Center, Shanghai, China, with a paired-end library. The reads were assembled into 28 contigs using VELVET (9). The genome annotation was performed by the RAST server (10). The G+C content was calculated by using the genome sequence.

The draft genome sequence of strain B1 is comprised of 2,942,988 bases with a G+C content of 67.5%. The genome was predicted to contain 2,789 predicted coding sequences (CDSs) together with 51 RNAs. A total of 351 subsystems were determined using the RAST server in the genome, and the information was used to construct the metabolic network by the RAST system. According to the annotation, we have predicted 70 CDSs related to stress response, among which 42 CDSs are responsible for oxidative stress response. Ten CDSs responsible for butanol biosynthesis and 14 CDSs responsible for acetyl-CoA fermentation to butyrate were annotated. Moreover, there are 46 CDSs that have been annotated as antibiotics and toxic compound-resistance genes, while genes related to virulence, disease, and defense were not found. Further investigation of the genome sequence could provide further insights into the mechanism of high-yield H_2_ production in strain B1.

### Nucleotide sequence accession numbers.

This whole-genome shotgun project has been deposited at DDBJ/EMBL/GenBank under the accession no. JHOF00000000. The version described in this paper is the first version, JHOF01000000.
